# Failure and multiple failure for disease modifying antirheumatic drugs in rheumatoid arthritis: Real-life evidence from a tertiary referral center in Italy

**DOI:** 10.1371/journal.pone.0281213

**Published:** 2023-02-02

**Authors:** Paola Conigliaro, Arianna D’Antonio, Luca D’Erme, Giulia Lavinia Fonti, Paola Triggianese, Alberto Bergamini, Maria Sole Chimenti

**Affiliations:** 1 Department of “Medicina dei Sistemi”, Rheumatology, Allergology and Clinical Immunology, Università di Roma “Tor Vergata”, Rome, Italy; 2 Institute of Radiology, Fondazione Policlinico Universitario Agostino Gemelli IRCCS, Rome, Italy; National Hospital Organization Kumamoto Saishun Medical Center, JAPAN

## Abstract

**Background:**

Rheumatoid Arthritis (RA) is a chronic inflammatory disease with a heterogeneous treatments’ clinical response. Goals of treatment are remission and low disease activity, which are not achieved in all patients despite the introduction of early treatment and the treat to target strategy.

**Objective:**

To investigate the causes of disease-modifying antirheumatic drugs (DMARDs) discontinuation and treatment failure and multiple failure for inefficacy, and to identify possible failure predictors’ according to RA patient characteristics in a real-world setting.

**Methods:**

718 RA patients were retrospectively evaluated. Conventional synthetic (cs) and biologic (b)DMARDs treatments line/s, effectiveness, and reasons of discontinuations were evaluated. Patients failing to at least two csDMARDs or bDMARDs’ drug for inefficacy were defined “csDMARDs multifailure” and “bDMARDs multifailure”, respectively. Discontinuation of at least two cs- and bDMARDs was termed “global multifailure”.

**Results:**

In total, 1422 csDMARDs and 714 bDMARDs treatment were analysed. Causes of csDMARDs discontinuation were intolerance (21.8%), inefficacy (20.2%), acute adverse reactions (5.3%) and severe infections (0.6%) while csDMARDs multifailure for inefficacy was observed in 5.7% of cases. Reasons of bDMARDs withdrawal were inefficacy (29%), intolerance (10.0%), acute adverse reaction (6.3%) and severe infections (1.5%). Altogether, 8.4% of patients were bDMARDs multifailure for inefficacy while 16.6% were global multifailure. Longstanding disease (≥ 12 months) and smoke habit, resulted as positive predictor of csDMARDs failure (OR 2.6 and OR 2.7, respectively). Thyreopathy was associated with both csDMARDs failure and global multifailure (OR 2.4 and OR 1.8, respectively). Higher prevalence of failure to at least one bDMARDs and global multifailure was detected in female than male (OR 2.3 and OR 2, respectively).

**Conclusions:**

Different causes of drug discontinuation were observed on DMARDs treatments. Demographic and clinical features were identified as possible predictors of both cs- and bDMARDs treatment failure and multiple failure, underlining the need of a more personalized therapeutic approach to achieve treatment targets.

## Introduction

Rheumatoid Arthritis (RA) is a chronic autoimmune systemic inflammatory disease that principally affects joints but may involve many organs and tissues. Extra-articular involvement and associated comorbidities play a key role in terms of prognosis and patient management. With the advent of biologic agents, the ultimate goals for treatment of RA patients are remission and low disease activity [1]. However, many patients do not reach or maintain these targets, in part due to the severity of the disease. In clinical practice a significant percentage of RA patients fail to respond to one or multiple conventional synthetic (cs) disease-modifying antirheumatic drugs (DMARDs) and biological/targeted synthetic (b/ts) DMARDs. Two‐thirds of patients receiving TNFα inhibitor (TNFi) failed to respond after 6 months of treatment [2] and approximately 6% of patients who started TNFi as their first bDMARD were subsequently refractory to multiple bDMARDs [2, 3]. However, uniform terminology and an appropriate definition of multifailure RA is still lacking. Recently, EULAR has proposed a definition for difficult-to-treat (D2T) RA, defined as patients who failed at least two b/tsDMARDs with different mechanisms of action after failing csDMARD therapy. Additionally, patients should have signs and/or symptoms suggestive of active disease, which is perceived as problematic by the patient and/or rheumatologist [4]. Different elements and modifiable lifestyle factors can contribute to the persistence of these signs and/or symptoms. Numerous authors have tried to identify predictors of response to bDMARDs but few analyses have been conducted to study which characteristics could made RA patients most susceptible of multiple failure to cs and bDMARDs [5].

The aim of the present study is therefore to investigate in a tertiary rheumatologic referral center the causes of cs and bDMARDs discontinuation and describe the characteristic of RA patients who fail to single and multiple drugs identifying possible predictors of failure.

## Materials and methods

A monocentric retrospective cross-sectional observational study, from 1 January 2009 to 31 December 2019, was conducted on 719 patients with established Rheumatoid Arthritis (RA) referred to the tertiary referral center in Italy Policlinico “Tor Vergata” of Rome in Italy.

Inclusion criteria were: 1) diagnosis of RA according to the 2010 European League Against Rheumatism response (EULAR)/ American College of Rheumatology (ACR) revised criteria for RA; 2) age ≥ 18 years old; 3) demographic and clinical data available from medical records; 4) regular follow-up with at least two consecutive visits every 3 months at the arthritis clinic of the Policlinico "Tor Vergata" in Rome [6].

The following data were collected from the patients’ records: demographic features, gender, age at RA onset, duration of the disease at the time of the study (in years -yrs), smoking habits (current smoker/non-smoker), extra-articular manifestation according to Young and Koduri and severity according to Malmö criteria, comorbidities in accordance with the classification of diseases described by Charlson including major adverse cardiovascular events (MACE), Pulmonary embolism/deep vein thrombosis (PE/DVT), history of thyroid diseases (including Hashimoto’s thyroiditis and Graves’ disease) and fibromyalgia, disease activity measured by Clinical Disease Activity Index (CDAI) and Disease Activity Score on 28 joint count C-reactive protein based (DAS28-CRP) [7–13].

Information on medical treatment at time of visit, such as previous therapies with cs and bDMARDs and reason of discontinuation were also recorded.

A disease duration < 12 months was defined as early arthritis, whereas a RA disease duration ≥ 12 months was arbitrarily defined as established arthritis [14].

The reasons for drug discontinuation included inefficacy, intolerance, acute adverse reaction, development of severe infections and non-specified cause (unknown). Acute adverse reaction was an adverse reaction, including all allergic-like and various physiologic reactions, which occurred within 1 hour after the administration of drug, as well as events which required hospital admission or caused death. Severe infection was defined as an infection requiring intravenous antibiotics, oxygen, pressors and/or fluid, led to hospitalisation, or causing death. Inefficacy was arbitrarily defined as lack or loss of efficacy, in terms of achievement the treatment targets of remisson or low disease activity, for at least 3 consecutive months, and requiring a treatment adjustment with switch or swap to another cs-/bDMARDs [4]. Patients who had at least two discontinuations due to inefficacy in csDMARDs or bDMARDs’ treatment were considered as “csDMARDs multifailure” and “bDMARDs multifailure”, respectively. Discontinuation of at least two cs- and two bDMARDs was defined as “global multifailure”.

Laboratory data were collected from clinical records and included rheumatoid factor (RF) (normal value < 20 IU/L), quantified by nephelometry using Immage 800® (Beckman Coulter, Fullerton, CA, USA), according to the manufacturer’s guidelines, and anti-citrullinated peptide antibodies (ACPA) (normal value < 20 IU/L), quantified by second generation commercial enzyme-linked immunosorbent assay (ELISA) kit (QUANTA Lite® CCP IgG, MedicalTechnology Promedt Consulting, St. Ingbert, Germany). Patients were considered seropositive if they showed a positivity for either RF or ACPA.

The study was performed in accordance with the Helsinki Declaration of 1975, as revised in 1983. All patients provided written informed consent and the local ethics committee approved the study.

## Statistical analysis

All data were stored on a server and statistical analyses was performed using GraphPad Prism version 6 (GraphPad software) and SPSS software version 24 for Windows (SPSS Inc., Chicago, IL, USA). To test normality of data sets the D’Agostino–Pearson omnibus test was used. Normally distributed variables were expressed as mean ± standard deviation (SD). Data for continuous variables are presented as the median with interquartile range (IQR), and variables were compared using the parametric unpaired T test or the non-parametric Mann–Whitney U test when appropriate. Categorical variables were compared using the Chi-squared test or Fisher’ exact test when appropriate. The significance of any correlation was determined by Spearman’s rank correlation coefficient. P values <0.05 with confidence interval (CI) 95% were considered statistically significant for all statistical evaluations. A multivariable logistic regression analysis was used to correct the p-value for gender, age, smoke, early arthritis, ACPA/RF positivity, extra-articular manifestations and comorbidities (Padj). Two-tailed P values less than 0.05 were considered statistically significant.

## Results

### Patients characteristics

The study included 718 patients with RA (77.4% female; mean age 63.9 ± 14.4 years) with mainly longstanding disease (n = 689 patients, 14.2 ± 9.5 years). RF and ACPA were detected in 72.4% (n = 520) and 67.1% (n = 482) of RA patients, respectively. Extra-articular manifestation showed a prevalence of 52.1% (n = 374) of which 18.6% (n = 134) of cases had a diagnosis of ILD. Most of RA patients had comorbidities (82.3%; n = 591) with hypertension being the most represented (46.2%, n = 332), followed by thyroid diseases (24.5%; n = 176). Clinical and laboratory features of the study population, including disease activity, are summarized in Table 1.

**Table 1:** Demographic and clinical data of rheumatoid arthritis patients.

	RA patients
	(n = 718)
Female gender, % (n)	77.4% (556)
Age (years)	63.9 ± 14.4
Observation period (years), median (IQR)	10 (7–10)
Disease duration (years)	14.2 ± 9.5
Early Arthritis % (n)	5.15% (37)
Current smokers, % (n)*	27.1% (160)
RF positive, % (n)	72.4% (520)
ACPA positive, % (n)	67.1% (482)
Comorbidities, % (n)	82.3% (591)
Hypertension, % (n)	46.2% (332)
Gastrointestinal disease, % (n)	20.2% (145)
Thyroid disorders, % (n)	24.5% (176)
Malignancy, % (n)	14.2% (102)
Kidney disease, % (n)	13.4% (96)
Diabetes mellitus, % (n)	10.7% (77)
MACE, % (n)	9.0% (65)
Fibromyalgia, % (n)	8.3% (60)
PE/DVT, % (n)	5.0% (36)
Extra-articular manifestations, % (n)	52.1% (374)
Severe, % (n)	51.3% (192)
Pleuritis, % (n)	4% (29)
Interstitial lung disease, % (n)	18.6% (134)
Pericarditis, % (n)	1.5% (11)
Mono/polyneuritis Multiplex, % (n)	0.6% (4)
Ulcers, % (n)	0.6% (4)
Episcleritis or scleritis, % (n)	1.2% (9)
Not severe, % (n)	48.7% (182)
Organizing pneumonia, % (n)	1.5% (11)
Arrhythmias, % (n)	4.5% (33)
Skin nodules, % (n)	5% (36)
Raynaud’s phenomenon, % (n)	1.8% (13)
Secondary Sjögren’s syndrome, % (n)	3.3% (24)
Sicca Syndome, % (n)	13.5% (91)
CDAI	8.7 ± 6.5
DAS28-CRP	4.3 ± 0.9

*Data not available for 128 patients.

Continuous values are shown as mean ± standard deviation or median (IQR) and categorical variables as absolute number and percentages. RA = rheumatoid arthritis; IQR = interquartile range; RF = rheumatoid factor, ACPA = anti-citrullinated peptide antibodies; MACE = Major adverse cardiovascular events; PE/DVT = Pulmonary embolism/deep vein thrombosis; CDAI = Clinical Disease Activity Index; DAS28-CRP = Disease Activity Score on 28 joint count C-reactive protein based.

All RA patients underwent at least one csDMARD (1.98 ±0.86 csDMARDs/ patient) and a total of 1422 csDMARD treatments were analyzed. Among csDMARDs, the most used was Methotrexate (42.8%, n = 609), followed by Hydroxychloroquine and Sulphasalazine (32.6%, n = 463 and 14.5%, n = 206, respectively). A percentage of 62.8% of patients (n = 451) were treated with at least one bDMARD (0.99 ± 1.04 bDMARDs/ patient) and a total of 714 bDMARDs treatments were examined. The study population treated with bDMARDs included 60.5% of patients (n = 273) at the first bDMARD, 26.9% (n = 121) at second line of bDMARD treatment and 12.6% (n = 57) at third or more lines of bDMARD. Combination therapy including one bDMARDs and MTX occurred in 48.5% (n = 346) of patients at median dose of MTX 13.7 ± 2.2 mg/week. Glucocorticoid were used in 52.1% of patients (n = 374) and at the median dose of 6.1 ± 1.8 mg/die of prednisone.

Among bDMARDs treatment, 82.6% was represented by TNFi (n = 590 treatment) and other bDMARDs were used in the 17.4% (n = 124) of cases. Treatment data are summarized in Table 2.

**Table 2:** cs- and bDMARD treatments in RA patients.

**Total of csDMARD treatments**	n = 1422
Methotrexate, % (n)	42.8 (609)
Hydroxychloroquine, % (n)	32.6 (463)
Sulfasalazine, % (n)	14.5 (206)
Leflunomide, % (n)	4.4 (63)
Cyclosporine, % (n)	5.7 (81)
**Total of bDMARD treatments**	n = 714
**TNFi, % (n)**	82.6 (590)
Etanercept, % (n)	40.9 (292)
Adalimumab, % (n)	32.1 (229)
Certolizumab, % (n)	6.7 (48)
Golimumab, % (n)	1.4 (10)
Infliximab, % (n)	1.5 (11)
**Other bDMARDs, % (n)**	17.4 (124)
Abatacept, % (n)	8.3 (59)
Tocilizumab, % (n)	5.9 (42)
Rituximab, % (n)	3.2 (23)

Data are expressed as absolute number and percentages. RA = rheumatoid arthritis; csDMARDs = conventional synthetic disease-modifying antirheumatic drugs; bDMARDs = biologic disease-modifying antirheumatic drugs; TNFi = tumor necrosis factor inhibitors.

### Treatment effectiveness/Treatment failure

Of 1422 csDMARDs treatments, 911 discontinuations were observed while 511 treatments were continued. Causes of discontinuation were represented by intolerance (310/1422, 21.8%), inefficacy (288/1422, 20.2%), acute adverse reactions (75/1422, 5.3%) and severe infections (8/1422, 0.6%) (Table 3). In the group of patients treated with csDMARDs, multifailure for inefficacy was observed in 5.7% of them (n = 41) (Table 3). Of 714 bDMARDs treatments, 433 discontinuations were observed. The main cause of bDMARDs withdrawal was inefficacy (207/714, 29.0%), following by intolerance (71/714, 10.0%), acute adverse reactions (45/714, 6.3%) and severe infections (11/714, 1.5%) (Table 3). In total, 8.4% of patients (n = 38) were bDMARDs multifailure due to inefficacy while 16.6% (n = 119) were global multifailure.

**Table 3:** Cause of cs- and bDMARDs discontinuation in RA treatments.

Drugs	Ongoing therapy	Inefficacy	Intolerance	Acute adverse reaction	Severe infection	Unknown
**csDMARDs**, % (n = 1422)	35.9 (n = 511)	20.2 (n = 288)	21.8 (n = 310)	5.3 (n = 75)	0.6 (n = 8)	16.2 (n = 230)
MTX, % (n = 609)	37.1 (n = 226)	23.2 (n = 141)	21.7 (n = 132)	1.8 (n = 11)	0.5 (n = 3)	15.7 (n = 96)
HCQ, % (n = 463)	38.2 (n = 177)	16.7 (n = 77)	16.8 (n = 78)	8.0 (n = 37)	0.0 (n = 0)	20.3 (n = 94)
SSZ, % (n = 206)	35.9 (n = 74)	17.0 (n = 35)	28.6 (n = 59)	4.9 (n = 10)	0 (n = 0)	13.6 (n = 28)
LEF, % (n = 63)	33.3 (n = 21)	46.0 (n = 29)	12.7 (n = 8)	8.0 (n = 5)	0 (n = 0)	0.0 (n = 0)
CYA, % (n = 81)	16.1 (n = 13)	7.4 (n = 6)	40.7 (n = 33)	14.8 (n = 12)	6.2 (n = 5)	14.8 (n = 12)
**bDMARDs, % (n = 714)**	39.3 (n = 281)	29.0 (n = 207)	10.0 (n = 71)	6.3 (n = 45)	1.5 (n = 11)	13.9 (n = 99)
**TNFi, %** (n = 590)	37.4 (n = 221)	31.7 (n = 187)	9.5 (n = 56)	5.9 (n = 35)	1.4 (n = 8)	14.1 (n = 83)
ETA, % (n = 292)	36.3 (n = 106)	37.7 (n = 110)	8.9 (n = 26)	4.1 (n = 12)	0.3 (n = 1)	12.7 (n = 37)
ADA, % (n = 229)	37.1 (n = 85)	27.9 (n = 64)	10.0 (n = 23)	5.3 (n = 12)	1.8 (n = 4)	17.9 (n = 41)
CERT, % (n = 48)	52.1 (n = 25)	10.4 (n = 5)	10.4 (n = 5)	14.5 (n = 7)	6.3 (n = 3)	6.3 (n = 3)
IFX, % (n = 11)	9.1 (n = 1)	54.5 (n = 6)	9.1 (n = 1)	18.2 (n = 2)	0 (n = 0)	9.1 (n = 1)
GOL, % (n = 10)	40.0 (n = 4)	20 (n = 2)	10 (n = 1)	20 (n = 2)	0 (n = 0)	10 (n = 1)
**Other bDMARDs, % (n = 124)**	48.4 (n = 60)	16.1 (n = 20)	12.1 (n = 15)	8.1 (n = 10)	2.4 (n = 3)	12.9 (n = 16)
ABA, % (n = 59)	55.9 (n = 33)	15.2 (n = 9)	10.2 (n = 6)	5.1 (n = 3)	0 (n = 0)	13.6 (n = 8)
TCZ, % (n = 42)	38.1 (n = 16)	14.3 (n = 6)	16.6 (n = 7)	14.3 (n = 6)	2.4 (n = 1)	14.3 (n = 6)
RTX, % (n = 23)	47.8 (n = 11)	21.8 (n = 5)	8.7 (n = 2)	4.3 (n = 1)	8.7 (n = 2)	8.7 (n = 2)

Data are expressed as absolute number and percentages. RA = rheumatoid arthritis; csDMARDs = conventional synthetic disease-modifying antirheumatic drugs; bDMARDs = biologic disease-modifying antirheumatic drugs; MTX = methotrexate; HCQ = Hydroxychloroquine; SSZ = sulfasalazine; LEF = leflunomide; CYA = cyclosporine; TNF-i = tumor necrosis factor inhibitors; ETA = etanercept; ADA = adalimumab; CERT = certolizumab pegol; IFX = infliximab; GOL = golimumab; ABA = abatacept; TCZ = tocilizumab; RTX = rituximab.

Among patients with inadequate response to one bDMARD 76.1% (n = 92) underwent a TNFi switch, whereas 23.9% (n = 29) experienced a swap. Likewise, among patients who have failed at least two bDMARDs, 21% (n = 8) were subjected two or more TNFi, while 30% (n = 79) underwent a swap. As regards the total of treatments, a significant percentage of discontinuation in TNFi group compared to other bDMARDs (85.2% vs 14.8%) was detected.

### Factors predicting of csDMARDs failure

Some factors have been recognized as predictor of treatment failure. Patients with longstanding disease (n = 681) were more likely to fail at least one csDMARD compared with those with early arthritis patients (n = 37) (17.1% vs 34.6%), as confirmed by multivariable analysis (Padj = 0.03, OR = 2.4, CI = 0.9–6.5).

From univariable analysis, the rate of csDMARDs multifailure in patients with extra-articular manifestation (8.0%, n = 30) was higher compared to patients without these manifestation (3.2%, n = 11) (p = 0.005, OR = 2.7). In particular, the prevalence of csDMARDs multifailure was 10.6% (n = 13) in patients with interstitial lung disease (ILD) compared to 4.7% in those without lung involvement (n = 28) (p = 0.01, OR = 1.4). However, these data were not confirmed in multivariable analysis.

Moreover, analysis showed an increase prevalence of csDMARDs multifailure in smokers (n = 13) compared to not smokers (n = 16) (9.6% vs 4.2%), confirmed by multivariable analysis (Padj = 0.01, OR = 2.7, CI = 1.2–5.8).

In addition, in patients having at least one comorbidity (6.6%, n = 39) higher proportion of csDMARDs multifailure was shown compared to remaining population (1.6%, n = 2) (p = 0.02, OR = 4.4). This result was not confirmed by multivariable regression.

Thyreopathy was the comorbidity most related to treatment failure. The prevalence of failure at least one csDMARD was higher in patients with thyroid disorders (43.2%, n = 76) compared to those without thyreopathy (30.8%, n = 167) (p = 0.008, OR = 1.6). Likewise, csDMARDs multifailure was greater in thyreopathics (8.5%, n = 15) than patients without thyroid diseases (4.2%, n = 26) (Padj = 0.001, OR 2.4, CI 1.2–5.0) (Table 4).

**Table 4:** Predicting factor of RA failure treatment.

	csDMARDs treatment							
			Failure <2			Multifailure		
Variable	Univariable		Multivariable*		Univariable		Multivariable*	
	OR	P	OR (95% CI)	P	OR	P	OR (95% CI)	P
Female gender	1.1	0.6	0.9 (0.6–1.4)	0.6	0.9	0.8	0.9 (0.4–2.3)	0.8
Longstanding RA	**2.56**	**0.03**	**2.4 (0.9–6.5)**	**0.03**	0.9	0.9	0.9 (0.2–4)	0.9
Smoke	1.1	0.5	1.1 (0.7–1.7)	0.5	**2.4**	**0.02**	**2.7 (1.2–5.8)**	**0.01**
RF positive	0.8	0.3	1 (0.5–1.9)	0.9	0.5	0.2	0.5 (0.1–2.1)	0.3
ACPA positive	1.1	0.4	1 (0.6–1.7)	0.9	1.3	0.4	1.9 (0.5–7.4)	0.2
EA manifestations	1.3	0.09	1.1 (0.7–1.6)	0.6	**2.65**	**0.005**	2.3 (0.9–5.6)	0.06
Comorbidities	1.2	0.2	1.3 (0.7–2.2)	0.3	**4.45**	**0.02**	NA	0.9
Hypertension	1.2	0.2	1.2 (0.8–1.7)	0.3	1.2	0.2	0.7 (0.4–1.4)	0.3
Thyreopathy	**1.59**	**0.008**	**2.4 (1.2–5.0)**	**0.001**	**1.85**	**0.06**	1.2 (0.5–3)	0.6

* adjusted for gender, age, smoke, early arthritis, ACPA/RFpositivity, extra-articular manifestations and comorbidities.

RA = rheumatoid arthritis; csDMARDs = conventional synthetic disease-modifying antirheumatic drugs; bDMARDs = biologic disease-modifying antirheumatic drugs; OR = odds ratio; CI = confidence interval; RF = rheumatoid factor; ACPA = anti-citrullinated peptide antibodies; EA = extra-articular manifestations.

### Factors predicting of bDMARDs failure

Demographic and clinical factors were explored for their influence on response to bDMARDs treatment. Failure to at least one bDMARD was higher in female (24.7%, n = 137) than male (13.4%, n = 22) (Pajd = 0.006, OR = 2.3, CI = 1.3–4.3). Analysing etanercept and adalimumab treatment, more failure was observed in female than males. In particular, females were more likely to fail etanercept [16.9% (n = 94) vs 10.4% (n = 17), p = 0.0422, OR = 1.8]. Compared with males, almost three times as many females failed adalimumab [10.6% (n = 59) vs 3% (n = 5), p = 0.0027, OR = 3.4]. From multivariable analysis, greater prevalence of failure was detected in female gender treated with adalimumab compared to male (Pajd = 0.41, OR = 2.9, CI 1.0–8.0). By contrast, etanercept has shown more effectively than others bDMARDs in patients with extra-articular manifestations (12.4% vs 18.1%, p = 0.0042, OR = 0.7).

As regard to comorbidities, greater prevalence of failure at least one bDMARD was observed in patients with thyroid disorders (27.8%, n = 49) compared to those without thyreopathy (20.2%, n = 110) (p = 0.0352, OR = 1.5, respectively). This data was not confirmed by multivariable analysis and no correlation with other factors was observed (Table 5).

**Table 5:** Predicting factor of RA failure treatment.

			bDMARDs treatment					
Variable		Failure <2				Multifailure		
	Univariable		Multivariable*		Univariable		Multivariable*	
	OR	P	OR (95% CI)	P	OR	P	OR (95% CI)	P
**Female gender**	**2.1**	**0.002**	**2.3 (1.3–4.3)**	**0.0006**	1.6	0.2	2 (0.7–6.4)	0.1
**Longstanding RA**	1.4	0.4	1.4 (0.5–3.6)	0.4	2.1	0.7	2.1 (0.2–16.1)	0.5
**Smoke**	0.9	0.7	1 (0.6–1.7)	0.8	1.3	0.5	1.5 (0.6–3.6)	0.3
**RF positive**	0.9	0.7	0.9 (0.4–1.8)	0.8	1.2	0.6	0.3 (0.1–1)	0.06
**ACPA positive**	0.9	0.8	1 (0.5–2)	0.8	0.9	0.8	1.5 (0.4–4.9)	0.4
**EA manifestations**	0.7	0.06	0.7 (0.4–1.1)	0.1	0.8	0.5	0.7 (0.3–1.7)	0.5
**Comorbidities**	0.7	0.2	0.7 (0.4–1.3)	0.3	0.7	0.3	0.8 (0.3–2.1)	0.7
**Hypertension**	0.7	0.1	1 (0.7–1.6)	0.8	0.7	0.3	1.1 (0.5–2.3)	0.8
**Thyreopathy**	**1.52**	**0.03**	1.4 (0.9–2.1)	0.09	1.6	0.1	1.1 (0.7–3.6)	0.7

* adjusted for gender, age, smoke, early arthritis, ACPA/RF positivity, extra-articular manifestations and comorbidities.

RA = rheumatoid arthritis; csDMARDs = conventional synthetic disease-modifying antirheumatic drugs; bDMARDs = biologic disease-modifying antirheumatic drugs; OR = odds ratio; CI = confidence interval; RF = rheumatoid factor; ACPA = anti-citrullinated peptide antibodies; EA = extra-articular manifestations.

### Factors predicting of global multifailure failure

Analysing global multifailure patients (n = 119), higher prevalence of female (18.1%, n = 101) was detected compared to male (11.1%, n = 18) (Pajd = 0.025, OR = 2, CI = 1.1–3.7).

Moreover, higher rate of global multifailure was detected in ACPA positive patients (18.5%, n = 91) compared with those negative for these antibodies (12.5%, n = 28) (p = 0.039, OR = 1.6). These data were not confirmed by multivariable analysis.

Higher prevalence of global multifailure was detected in patients with thyreopathy (23.3% n = 42) compared to those without thyroid disorders (14.4%, n = 77) (Padj = 0.007, OR = 1.8, CI = 1.2–2.9) (Table 6).

**Table 6:** Predicting factor of RA failure treatment.

		Global multifailure		
Variable	Univariable		Multivariable*	
	**OR**	**P**	**OR (95% CI)**	**P**
**Female gender**	**1.71**	**0.034**	**2.02 (1.1–3.7)**	**0.02**
**Longstanding RA**	0.8	0.8	0.8 (0.2–3.9)	0.8
**Smoke**	**1.6**	**0.04**	1 (0.9–1)	0.5
**RF positive**	0.7	0.2	1 (0.3–3.2)	0.9
**ACPA positive**	**1.61**	**0.039**	1.2 (0.4–3.5)	0.7
**EA manifestations**	1.1	0.5	0.7 (0.4–1.5)	0.4
**Comorbidities**	1.2	0.5	2 (0.9–3.1)	0.06
**Hypertension**	1.1	0.5	0.6 (0.3–1.3)	0.2
**Thyreopathy**	**1.81**	**0.0056**	**1.8 (1.2–2.9)**	**0.007**

* adjusted for gender, age, smoke, early arthritis, ACPA/RF positivity, extra-articular manifestations and comorbidities.

RA = rheumatoid arthritis; csDMARDs = conventional synthetic disease-modifying antirheumatic drugs; bDMARDs = biologic disease-modifying antirheumatic drugs; OR = odds ratio; CI = confidence interval; RF = rheumatoid factor; ACPA = anti-citrullinated peptide antibodies; EA = extra-articular manifestations.

## Discussion

Treatment options for inflammatory arthritis have expanded greatly with the availability of different bDMARDs and more recently tsDMARDs [15]. However, the management of arthritis may be difficult because of several factors associated to the patient such as age, gender, life-style habits or related to the disease itself as the presence of negative prognostic factors, autoantibodies, extra- articular manifestations, or comorbidities [7–18]. It is also well-known that a first treatment failure may increase the probability to further treatment failures. The proportion of patients refractory to one or multiple cs and bDMARDs extrapolated from randomised controlled trial and real-life data suggests approximately 20% progress onto different lines of treatment [19]. Recently, EULAR recommendations have been developed to define D2T patients and points to consider for the management of these patients [4]. However, there is not a unique definition of refractory RA that implies to be resistant to multiple treatments with persistent joint or systemic inflammation, features that confer a poor prognosis to the patient.

In our cohort the main reasons for csDMARDs discontinuation were intolerance and inefficacy with a similar rate, while only a small percentage of patients discontinued because of infections or acute adverse reactions. Only 6% of patients were multifailure for inefficacy. Indeed, csDMARDs side effects, such as mild gastrointestinal symptoms, may negatively impact quality of life and may cause loss of adherence [15]. Some factors seem to be associated with loss of efficacy, such as female sex or ethnicity, and sex differences on pharmacokinetic have been hypothesized [20]. Despite csDMARDs being the first line treatment in a previous analysis on 1287 arthritic patients, 34% of patients stopped MTX due to intolerability; therefore, the identification of risk factors associated to intolerability should be further investigated since MTX represents the anchor drug in the treatment of RA [21].

Causes of bDMARDs discontinuations were also examined and the main cause in our cohort was inefficacy in 29% of cases followed by intolerance in 10% of cases suggesting an overall better tolerance of bDMARDs compared with csDMARDs. In the entire population we identified 8% of patients being bDMARDs multifailure and 16% of cases of global multifailure.

Previous studies have highlighted that switching between TNFi is less effective than swapping to drugs with different mechanisms. Given these assumptions, these patients covered by the concept of D2T [4]. Consistent with literature, our results have shown a higher percentage of discontinuation in patients undergoing TNFi switch than those experiencing swap.

We identified few factors associated to csDMARDs inefficacy such as having longstanding disease, smoking habit, the presence of extra-articular manifestations as ILD, and comorbidities as thyreopathy. Some of these factors as thyreopathy together with female sex were associated also to bDMARDs failure and being global multifailure.

Longstanding disease has been associated with a worse prognosis due to chronic inflammation leading to joint and extra-articular damage in terms of bone erosions and fragility, sarcopenia and the occurence of comorbidities [22–24]. However, a clear definition of established RA is missing being crucial in these patients that are often at high risk to be resistant to treatment and may be difficult for the management. Therefore, clinical and molecular studies are warranted in this population to understand the immunopathological mechanisms behind treatment resistance.

Other factors as smoke and ILD were previously identified as negative prognostic factors although EULAR recommendations do not consider them as traditional negative prognostic factors. Indeed, smoking has been recognized as a predictor of bone erosions in a cohort of early RA and we confirm that smoke habit is a negative prognostic factor predictor of csDMARDs multiple failure [25]. ILD is the most common lung manifestation of RA and the second most common cause of mortality. However, it is underestimated because it is often paucisymptomatic [26]. Early recognition of lung involvement might reduce the progression to lung fibrosis and be associated with and early introduction of a bDMARD or an optimal therapeutic regimen limiting some csDMARDs as leflunomide that could be detrimental for lung involvement [27]. In our cohort ILD has a prevalence of 18% and seems to be associated with a worse prognosis in terms of csDMARDs multiple failure, although this data was not confirmed in the multivariable analysis possible reflecting the heterogeneity of enrolled patients in a real-life retrospective cohort.

Patients admitted in our hospital have a high prevalence of extra-articular manifestations and comorbidities reflecting the admittance to a tertiary referral center and may be challenging in the therapeutic management. Among comorbidities, in our cohort thyreopathy has shown a prevalence of 24.5% and was associated with an increased risk to fail at least one csDMARD or one bDMARD, being multifailure for csDMARDs or global multifailure. Studies from the literature on the prevalence of thyroid disorders in RA show a high variability ranging from 1.7% to 32.3% depending on ethnicity, environmental differences (iodine intake) and criteria for diagnosis [28]. A negative impact of thyroid disorders on RA has been previously hypothesized, although it remains controversial. Some Authors suggested that thyroid dysfunction is associated with ACPA positivity and poor response [29–31]. A recent study explored the negative impact of autoimmune thyroid disease on RA disease activity and response to MTX [32]. In particular, in line with our results, thyroid disease negatively influenced the overall chance to achieve EULAR response at 3 and 6 months. These data need to be further investigated aiming to dissect the relative contribution of both thyroid disorders and anti- thyroid antibodies on RA disease activity.

Female sex is a well-known negative prognostic factor. In our cohort it was associated with failure to at least one bDMARD; these data were confirmed both in those patients that received adalimumab and etanercept, and also in global multifailure. In a previous analysis in a small cohort of RA patients, male sex was identified as a factor predicting 2-years remission and LDA in patients treated with TNF-inhibitors [33]. Moreover, in a recent analysis sex and disease duration predicted the achievement of remission and LDA in three trials (ASPIRE, ATTRACT and GO-BEFORE) [34]. Likewise, in a study on 4268 RA patients, male sex was associated with longer drug survival on bDMARDs, confirming gender as an important predicting factor of bDMARD response [35].

The study has some limitations such as the retrospective design, missing data and the great variety of patient characteristics and treatments which can lead to possible bias and miss- classification, compared to randomized controlled trials. In addition, retrospective studies may need very large sample sizes for rare outcomes, like the presence of extra-articular manifestations, and subgroup of patients with different treatments. Moreover, in our study we did not include the time to event analysis, nevertheless time to exposure and drug survival would provide more extensive results. However, the real-world setting is a strength which allowed to identify multiple predicting factors of treatment failure to cs and bDMARDs. Some of these factors may be modified as smoking habit while others may be recognized early with periodic screening for extra-articular manifestations and comorbidities in order to minimize failure and multiple failure to DMARDs.

## Supporting information

S1 Data (XLSX)
